# Efficacy of lenvatinib combined with sintilimab and transcatheter arterial chemoembolization in conversion therapy for unresectable hepatocellular carcinoma

**DOI:** 10.12669/pjms.42.9.16251

**Published:** 2026-09

**Authors:** Jianwu Long, Jinli Zheng, Shiyi Chen, Peng Ou, Xianzhou Lu

**Affiliations:** 1 Jianwu Long, Department of Hepatobiliary Surgery, The Affiliated Nanhua Hospital, Hengyang Medical School, University of South China, Hengyang, Hunan Province 421002, P.R. China; 2 Jinli Zheng, Department of Hepatobiliary Surgery, The Affiliated Nanhua Hospital, Hengyang Medical School, University of South China, Hengyang, Hunan Province 421002, P.R. China; 3 Shiyi Chen, Department of Hepatobiliary Surgery, The Affiliated Nanhua Hospital, Hengyang Medical School, University of South China, Hengyang, Hunan Province 421002, P.R. China; 4 Peng Ou, Department of Hepatobiliary Surgery, The Affiliated Nanhua Hospital, Hengyang Medical School, University of South China, Hengyang, Hunan Province 421002, P.R. China; 5 Xianzhou LuThe Affiliated Nanhua Hospital, Department of Hepatobiliary Surgery, Department of Hepatobiliary Surgery, The Affiliated Nanhua Hospital, Hengyang Medical School, University of South China, Hengyang, Hunan Province 421002, P.R. China

**Keywords:** Conversion therapy, Lenvatinib, Sintilimab, Transcatheter Arterial Chemoembolization (TACE), Unresectable Hepatocellular Carcinoma

## Abstract

**Objectives:**

To compare the efficacy of lenvatinib combined with sintilimab versus transcatheter arterial chemoembolization (TACE) in the conversion therapy for unresectable hepatocellular carcinoma (HCC).

**Methodology:**

A retrospective analysis of the clinical data of 153 patients with unresectable HCC admitted to the Affiliated Hospital of Nanhua University from January 2023 to December 2024 was performed. According to the treatment regimens, the patients were divided into two groups: the study group (n=79) treated with lenvatinib plus sintilimab and the control group (n=74) treated with TACE. The efficacy of conversion therapy, tumor marker levels, tumor morphological features, blood supply, and adverse reactions were compared between the two groups.

**Results:**

Compared with those in the control group, the objective response rate (ORR) (67.09% vs. 47.30%, p=0.013), disease control rate (DCR) (91.14% vs. 79.73%, p=0.044), and conversion resection rate (48.10% vs. 28.38%, p=0.012) in the study group were significantly greater. Similarly, compared with the control group, the study group also exhibited lower levels of alpha-fetoprotein (AFP) (245.76±108.54 ng/mL vs. 186.42±95.37 ng/mL, p<0.0001), protein induced by vitamin K absence or antagonist II (PIVKA-II) (309.25±96.78 ng/mL vs. 245.68±82.35 ng/mL, p<0.0001), and chemokine (C-C motif) ligand 2 (CCL2) (71.76±23.81 ng/mL vs. 58.36±18.42 ng/mL, p<0.0001). Additionally, the blood flow signal intensity of tumor-feeding arteries (t=5.620; p<0.001), reduction rate of maximum tumor diameter (t=3.436; p<0.001), and tumor necrosis rate (t=7.025; p<0.001) were significantly better in the study group than in the control group. There was no significant difference in the incidence of adverse reactions, including hand-foot skin reactions (p=0.474), gastrointestinal reactions (p=0.875), liver function injury (p=0.672), myelosuppression (p=0.539), or fever (p=0.392), between the two groups (P>0.05).

**Conclusion:**

Compared with TACE, lenvatinib combined with sintilimab can improve the therapeutic efficacy of conversion therapy for unresectable HCC, with comparable safety.

## INTRODUCTION

Primary liver cancer has one of the highest incidences and mortality rates worldwide.^1^ Hepatocellular carcinoma (HCC), the most common primary liver cancer, accounts for approximately 85–90% of all cases.^2^ Owing to its insidious onset and rapid progression, most patients are diagnosed at intermediate or advanced stages, complicated by vascular invasion, intrahepatic metastasis, or distant metastasis, and are therefore ineligible for radical surgery.^3^,^4^ Transcatheter arterial chemoembolization (TACE), a classic local treatment modality for unresectable HCC, can achieve tumor shrinkage by embolizing the tumor blood supply and locally releasing chemotherapeutic drugs.^5^,^6^ However, TACE also faces significant challenges, including local recurrence due to incomplete tumor necrosis, heterogeneous drug distribution, tumor angiogenesis induced by ischemia and hypoxia, postembolization syndrome, limited efficacy in long-term and advanced HCC, and a high risk of disease progression.^7^–^9^

Recent advances in targeted and immunotherapies have enabled the development of conversion therapy, a treatment strategy that uses systemic or local therapies to shrink unresectable advanced tumors, potentially enabling surgery or transplantation.^10^ Lenvatinib, a tyrosine kinase inhibitor, inhibits multiple targets involved in tumor angiogenesis and proliferation, including vascular endothelial growth factor (VEGF) receptors and fibroblast growth factor receptors (FGFRs).^11^–^13^ Lenvatinib has emerged as a critical first-line systemic therapy against HCC, particularly for patients with advanced or unresectable disease who are not candidates for surgical resection or locoregional interventions.^14^ However, several challenges, including acquired resistance caused by tumor vascular adaptation, the need for biomarker-driven patient selection, adverse events (such as hypertension, proteinuria, and diarrhea), and heterogeneous efficacy across etiologies, exist.^15^-^17^ To overcome these limitations, combinative strategies have been developed. For example, in a retrospective analysis of 345 patients who received HCC and TACE treatment, Ni et al.^18^ suggested that the combination of lenvatinib and programmed death receptor-1 (PD-1) inhibitors achieved a better objective response rate and significantly improved the progression-free survival of patients (compared with those who received TACE and lenvatinib treatment). Sintilimab, a programmed death receptor-1 (PD-1) inhibitor developed in China, restores antitumor immune function by blocking the immune checkpoint pathway and has demonstrated efficacy in sustained tumor suppression.^19^ However, the use of sintilimab is limited by immune-related adverse events, including hepatitis and endocrinopathies, and vigilance monitoring is needed, particularly in patients with underlying cirrhosis.^20^

Studies have shown that combined targeted therapy and immunotherapy have synergistic antitumor effects, significantly improving the objective response rate in unresectable HCC, and are emerging as important regimens for clinical conversion therapy.^21^,^22^ However, the efficacy of lenvatinib combined with sintilimab versus TACE in the treatment of unresectable HCC is still unclear. This study aimed to assess the therapeutic effects of the two regimens. These results may provide precise, practical evidence to guide clinical treatment strategies for unresectable HCC, optimize diagnosis and treatment protocols for intermediate- and advanced-stage HCC, and offer new clinical insights to improve patient prognosis.

## METHODOLOGY

This retrospective analysis included the clinical data of 153 patients with unresectable HCC admitted to the Affiliated Hospital of Nanhua University from January 2023 to December 2024. Patients were divided into groups by treatment regimen: the study group comprised 79 patients treated with lenvatinib plus sintilimab, and the control group comprised 74 patients treated with TACE.

### Ethical approval:

The ethics committee of our hospital approved this study (number 2022-ky-269; date: November 15, 2022).

### Inclusion criteria:

- Conformed to the diagnostic criteria for HCC.^23^
- Diagnosis confirmed by histopathological examination or a combination of clinical imaging (enhanced CT/MRI) and alpha-fetoprotein (AFP) detection.
- Classified as the Barcelona Clinic Liver Cancer (BCLC) stages B or C.^24^
- Inoperable for radical resection (single tumor diameter > 5 cm, multiple tumors with maximum diameter > 3 cm, complicated with vascular invasion or intrahepatic metastasis, and without indications for radical surgery).
- Child-Pugh grade A or B.
- Expected survival time ≥ 3 months.
- Complete clinical data available.

### Exclusion criteria:

- Complicated with other malignant tumors.
- Complicated with severe infections (e.g., active hepatitis, sepsis), coagulation disorders or bleeding tendency (e.g., gastrointestinal ulcer bleeding)
- A history of radical hepatectomy, targeted therapy, immunotherapy or TACE for HCC;
- Presence of contraindications to the relevant treatments.
- Complicated with renal failure, decompensated cirrhosis or other severe complications affecting treatment and follow-up.
- Hypersensitivity to the study drugs or chemotherapeutic agents used in TACE.

### Treatment approaches:

- ***Transcatheter arterial chemoembolization (TACE) regimen:*** One week before treatment, liver and kidney function tests, coagulation tests, an electrocardiogram, and other relevant examinations were performed. Enhanced CT/MRI was performed to clarify the feeding arteries, number, and size of the tumors. Fasting and water deprivation were implemented for six hours preoperatively, a routine iodine allergy test was conducted, and intravenous access was established. The Seldinger technique was adopted for femoral artery puncture and catheterization, with the catheter selectively inserted into the tumor-feeding artery. The catheter position was confirmed by digital subtraction angiography (DSA), followed by slow injection of chemoembolic agents (epirubicin 50 mg/m² + cisplatin 20 mg/m² + ultraliquid lipiodol 10–20 mL, with dosage adjusted according to tumor size) until complete embolization of the tumor-feeding artery and stagnation of blood flow were detected by DSA. After the operation, the puncture site was compressed for 15–20 minutes, followed by pressure bandaging and bed rest for 24 hours. Vital signs and abdominal symptoms were closely monitored. Routine supportive treatments, including anti-infection (cephalosporin antibiotics), liver protection (polyene phosphatidylcholine), analgesia (parecoxib sodium), and fluid replacement, were administered. Enhanced CT/MRI was performed one month after surgery to evaluate tumor necrosis. TACE treatment was repeated every four to six weeks on the basis of patient tolerance and therapeutic efficacy, with a total of no more than six treatment cycles.
- ***Lenvatinib Combined with Sintilimab Regimen:*** Lenvatinib capsules were taken orally, with the initial dosage adjusted according to patient weight: 12 mg once daily for patients with a weight of ≥ 60kg and 8mg once daily for patients with a weight of < 60 kg. The medication was administered at a fixed time daily, either on an empty stomach or with food. Sintilimab was intravenously injected at a dosage of 200mg per session, with a treatment cycle of three weeks. The infusion time was ≥ 30 minutes, and the treatment was continued for four to six consecutive cycles.

### Outcome measures:

- ***Efficacy of conversion therapy:*** The efficacy was evaluated according to the Response Evaluation Criteria in Solid Tumors (RECIST) Version 1.1.^25^ All patients underwent routine follow-up every two to three treatment cycles. The collected clinical parameters included liver contrast-enhanced CT or MRI, electrocardiogram, laboratory test (including biochemical panel, complete blood count, coagulation profile, alpha-fetoprotein [AFP], and thyroid function tests), and clinical symptoms and signs. Changes in target lesions were assessed using enhanced CT/MRI and classified as complete response (CR), partial response (PR), stable disease (SD), or progressive disease (PD). The objective response rate (ORR) was calculated as \text{ORR} = \frac{\text{CR} + \text{PR}}{\text{Total number of cases}} \times 100\%, and the disease control rate (DCR) was calculated as \text{DCR} = \frac{\text{CR} + \text{PR} + \text{SD}}{\text{Total number of cases}} \times 100\%. The conversion resection rate (the proportion of patients who achieved radical surgical indications and underwent surgery) was recorded. Definition of successful transformation: After systemic transformation therapy, the tumor lesion significantly regressed, the boundary was clear, and there was no new distant metastases or large vessel invasion; imaging evaluation suggested that the lesion could be completely resected with R0, without extensive infiltration into the abdominal cavity or irreversible invasion of important organs. After multidisciplinary surgical evaluation, the indication for radical surgery has been reached, and radical surgical resection has been completed. The conversion resection rate is defined as the proportion of patients who meet the above conversion success criteria and complete radical surgery among the total enrolled patients.
- ***Serum tumor marker levels:*** Fasting venous blood samples (5 mL) were collected from patients and centrifuged at 3000 r/minutes for 10 minutes to separate the serum. The levels of alpha-fetoprotein (AFP), protein induced by vitamin K absence or antagonist II (PIVKA-II), and chemokine (C-C motif) ligand 2 (CCL2) were detected using an enzyme-linked immunosorbent assay (ELISA).
- ***Morphological and blood supply changes of tumor lesions:*** Quantitative assessment of tumor morphology was performed using contrast-enhanced CT or MRI. The evaluated parameters included the reduction rate of maximum tumor diameter, tumor necrosis rate (necrotic volume/total lesion volume × 100%), and blood flow signal intensity of the tumor-feeding arteries, which was graded as Grade 0 (no blood flow), Grade-1 (minimal blood flow), Grade-2 (moderate blood flow), or Grade-3 (abundant blood flow). The following adverse reactions were recorded, namely, hand-foot skin reactions (skin changes such as redness, peeling, blisters, or ulceration pain on the palms and soles after medication, excluding skin lesions caused by trauma, fungal infections, and preexisting skin diseases), gastrointestinal reactions (including drug-induced nausea, vomiting, diarrhea, oral mucosal inflammation, and other discomforts, excluding gastrointestinal symptoms caused by preexisting chronic gastrointestinal diseases, unclean diet, and intestinal obstruction), liver function damage (ALT, AST after medication), or increased total bilirubin levels compared with baseline, excluding basic liver and gallbladder diseases such as viral hepatitis, biliary tract disease, and autoimmune liver disease); bone marrow suppression (based on a decrease in white blood cells, neutrophils, hemoglobin, and platelets after medication, excluding abnormal blood count caused by primary hematological diseases, acute blood loss, and severe infections); and fever (temperature increase without clear evidence of bacterial or viral infection after medication, excluding tumor self-cancerous fever, various coinfections, and fever induced by autoimmune diseases), were recorded.
- ***Adverse reactions:*** The incidence of adverse reactions was documented for both groups.

### Statistical analysis:

Data analysis was performed using SPSS 25.0 software. Quantitative data are expressed as the mean ± standard deviation (\bar{x}\pm s). Following assessment of normality, normally distributed quantitative variables were compared using the independent-samples t test, whereas non-normally distributed variables were analyzed using the Mann–Whitney U test. Categorical variables were compared using the chi-square test. P < 0.05 was considered to indicate statistical significance.

## RESULTS

There were no statistically significant differences in baseline data, including age, sex, body mass index (BMI), disease duration, tumor diameter, BCLC stage, comorbidities, AFP level, or Child-Pugh grade, between the two groups (P>0.05). Table-I.

**Table-I:** Comparison of the general data between the two groups.

Items	Study Group(n=79)	Control Group(n=74)	t/χ^2^ value	P value
Age(*x̄*±*s*, years)	61.52±8.36	60.93±7.81	0.450	0.653
***Gender [n(%)]***				
Male	52(65.82)	46(62.16)	0.222	0.637
Female	27(34.18)	28(37.84)		
Body Mass Index(*x̄*±*s*, kg/m²)	24.09±2.35	24.41±2.28	0.853	0.395
Disease Course(*x̄*±*s*, months)	9.76±3.68	10.12±3.75	0.599	0.550
Tumor Diameter (*x̄*±*s*, cm)	7.89±2.19	8.21±2.26	0.889	0.375
***BCLC Stage [n(%)]***				
Stage B	33(41.77)	35(47.30)	0.472	0.492
Stage C	46(58.23)	39(52.70)		
***Comorbidities [n(%)]***				
Cirrhosis	45(56.96)	40(54.05)	0.131	0.718
Hypertension	30(37.97)	27(36.49)	0.036	0.849
Diabetes Mellitus	20(25.32)	17(22.97)	0.114	0.735
***AFP [n(%)]***				
≥400 ng/mL	38(48.10)	33(44.59)	0.189	0.664
<400 ng/mL	41(51.90)	41(55.41)		
***Child-Pugh Grade [n(%)]***				
Grade A	42(53.16)	41(55.41)	0.077	0.781
Grade B	37(46.84)	33(44.59)		

The ORR (67.09%), DCR (91.14%), and conversion resection rate (48.10%) in the study group were significantly greater than those in the control group (47.30%, 79.73%, 28.38%, respectively) (P < 0.05). Table-II.

**Table-II:** Comparison of Conversion Therapy Efficacy between the Two Groups [n(%)].

Group	No. of Cases	CR (%)	PR (%)	SD (%)	PD (%)	ORR (%)	DCR (%)	Conversion Resection Rate
Study Group	79	11(13.92)	42(53.16)	19(24.05)	7(8.86)	53(67.09)	72(91.14)	38(48.10)
Control Group	74	4(5.41)	31(41.89)	24(32.43)	15(20.27)	35(47.30)	59(79.73)	21(28.38)
*χ^2^value*		2.246	1.946	1.329	3.167	6.125	4.040	6.274
*P value*		0.1339	0.163	0.249	0.075	0.013	0.044	0.012

CR: complete response; PR: partial response; SD: stable disease; PD: progressive disease;

ORR: objective response rate (ORR); DCR: disease control rate.

There were no significant differences in the levels of AFP, PIVKA-II or CCL2 between the two groups before treatment (P>0.05). After treatment, the AFP, PIVKA-II, and CCL2 levels in both groups were lower than those before treatment, and those in the study group were significantly lower than those in the control group (P<0.05). Table-III).

**Table-III:** Comparison of tumor markers between the two groups (*x̄*±*s*, ng/mL).

Time Point	Group	No. of Cases	AFP	PIVKA-II	CCL2
Before Treatment	Study Group	79	689.35±215.62	896.73±212.45	128.54±35.62
	Control Group	74	693.58±208.45	875.36±198.72	130.76±33.89
	*t value*		0.123	0.641	0.394
	*P value*		0.902	0.522	0.694
After Treatment	Study Group	79	186.42±95.37	245.68±82.35	58.36±18.42
	Control Group	74	245.76±108.54	309.25±96.78	71.76±23.81
	*t value*		3.598	4.385	3.908
	*P value*		<0.001	<0.001	<0.001

There were no significant differences in the blood flow signal intensity of tumor-feeding arteries between the two groups before treatment (P>0.05). After treatment, the blood flow signal intensity of the tumor-feeding arteries in both groups was lower than that before treatment, and the intensity in the study group was significantly lower than that in the control group (P<0.05). In addition, the reduction rate of maximum tumor diameter and the tumor necrosis rate were significantly greater in the study group than in the control group (P<0.05). Table-IV). The incidence rates of hand-foot skin reaction, gastrointestinal reaction, liver function injury, myelosuppression, and fever were similar in both groups (P>0.05). (Table-V).

**Table-IV:** Comparison of tumor morphology features and blood supply between the two groups (*x̄*±*s*).

Time Point	Group	No. of Cases	Reduction Rate of Maximum Tumor Diameter(%)	Tumor Necrosis Rate(%)	Blood Flow Signal Intensity of Tumor-Feeding Arteries (Grade)
Before Treatment	Study Group	79	/	/	2.75±0.62
	Control Group	74	/	/	2.68±0.59
	*t value*		/	/	0.714
	*P value*		/	/	0.476
After Treatment	Study Group	79	58.36±15.72	79.54±8.36	0.89±0.45
	Control Group	74	50.18±13.56	70.89±6.72	1.36±0.58
	*t value*		3.436	7.025	5.620
	*P value*		0.001	<0.001	<0.001

**Table-V:** Comparison of adverse reactions between the two groups [n(%)].

Group	No. of cases	Hand-Foot Skin Reaction	Gastrointestinal Reaction	Liver Function Injury	Myelosup-pression	Fever
Study Group	79	21(26.58)	19(24.05)	16(20.25)	12(15.19)	11(13.92)
Control Group	74	16(21.62)	17(22.97)	13(17.57)	14(18.92)	7(9.46)
*χ^2^ value*		0.513	0.025	0.179	0.377	0.734
*P value*		0.474	0.875	0.672	0.539	0.392

## DISCUSSION

In patients with unresectable HCC who underwent TACE, compared with TACE, the combined lenvatinib/sintilimab regimen achieved superior therapeutic efficacy and did not increase the risk of adverse events.

The core goal of conversion therapy in HCC is to downstage tumors and eliminate micrometastatic lesions via highly effective therapeutic modalities.^26^ Despite being the gold standard of traditional local therapy, TACE still has significant limitations.^27^ Strategies have been developed to overcome those limitations in HCC treatment via technological, pharmacological, and combinational innovations. For example, drug-eluting bead TACE (DEB-TACE)^28^ improved drug delivery uniformity and reduced systemic toxicity. Balloon-occluded TACE (B-TACE) results in a better oncological response than drug-eluting-microsphere (DEM-TACE) does in patients with HCC because it prevents collateral flow.^29^ Cone-beam CT and 3D angiography enable precise tumor targeting, minimize nontumor embolization and preserve hepatic function.^30^,^31^ To address incomplete necrosis, TACE combined with systemic therapies, particularly antiangiogenic agents (sorafenib and lenvatinib) or immune checkpoint inhibitors, has emerged as a promising strategy to suppress residual tumor angiogenesis and enhance immune-mediated clearance.^32^,^33^ Furthermore, ongoing research into biodegradable embolic materials, nanoparticle-based drug carriers, and radiomics-guided patient selection aims to further refine TACE efficacy and expand its applicability across broader HCC stages.^34^,^35^ Notably, the combination of lenvatinib and PD-1 inhibitors (camrelizumab, tislelizumab, sintilimab, or pembrolizumab) is associated with a lower incidence of Grade-3 adverse events (less than 5%), suggesting that the combined strategy has a good safety profile for unresectable HCC.^36^

In this retrospective analysis, the efficacy of lenvatinib and sintilimab combination therapy (in the study group) and TACE alone (in the control group) was compared. We demonstrated that the ORR, DCR, and conversion resection rate in the study group were significantly greater than those in the control group (P<0.05). Similar results have been obtained by Han RY et al.^37^, who confirmed that compared with conventional TACE, the combined regimen has significantly greater conversion efficacy. Although the study by Zhao L et al.^38^ on advanced HCC did not focus on conversion therapy, the ORR of targeted therapy combined with immunotherapy was significantly greater than that of TACE, further corroborating the strong tumor-shrinking advantage of this combination regimen.

The underlying mechanism may involve synergistic antitumor effects exerted by the two agents. As a multitarget tyrosine kinase inhibitor, lenvatinib potently inhibits tumor angiogenesis mediated by angiogenic signaling pathways (such as VEGF and FGFR) and subsequently restrains the tumor nutrient supply. Lenvatinib also has potential roles in reversing the antitumor microenvironment through vascular normalization, reducing the infiltration of immunosuppressive cells, and creating favorable conditions for immunotherapy.^39^ On the other hand, sintilimab blocks the PD-1/PD-L1 pathway to relieve the inhibition of immune cells by tumor cells, activates T-cell-specific killing of tumor cells, and achieves systemic tumor control.^40^ While TACE can directly embolize tumor-feeding arteries to induce local necrosis, it lacks effective control over intrahepatic micrometastases and potential distant metastases.^41^ Moreover, the postoperative tumor ischemia-hypoxia environment can activate the hypoxia-inducible factor pathway, promote the release of angiogenic factors such as VEGF, accelerate the proliferation of residual tumor cells and the formation of collateral circulation, and ultimately compromise conversion efficacy.^41^

Dynamic changes in serum tumor markers are important quantitative indicators for evaluating therapeutic efficacy and tumor burden in patients with HCC. Li et al.^42^ determined the dynamic changes in serum AFP levels in HCC patients receiving TACE, hepatic arterial infusion chemotherapy (HAIC), molecular targeted therapies (MTTs), and immune checkpoint inhibitors (ICIs). They reported that AFP was a potential independent predictor of overall survival and progression-free survival. In addition, HCC patients with a log10 AFP ≥1.40 benefitted more from receiving lenvatinib-based triple therapy.^43^ Moreover, the AFP decrease ratio is an independent predictor of a lower recurrence risk in HCC patients receiving hepatic arterial infusion chemotherapy (HAIC), bevacizumab, and sintilimab.^44^ These studies suggest that determining AFP levels might also guide lenvatinib treatment. PIVKA-II is positively correlated with tumor burden and is also a predictor of TACE response in patients with HCC.^45^ The chemokine receptor CCR2, along with its ligand CCL2, plays an essential role in mediating tumor growth and angiogenesis by affecting the infiltration and activation of host immune cells.^46^ For example, the CCL2/CCR2 axis is involved in the recruitment of monocytes and macrophages to tumor sites, thus shaping the immunosuppressive TME of HCC.^47^ In this study, the levels of AFP, PIVKA-II, and CCL2 decreased after treatment, with a greater reduction observed in the study group (P<0.05). These results are consistent with the research trend reported by Zhao FY et al.^48^, further corroborating the potent tumor-killing effect of the combined treatment. AFP and PIVKA-II are specific markers for HCC, and their levels are directly correlated with tumor proliferative activity.^49^ The significant reduction in these two markers indicates massive apoptosis and suppressed tumor cell proliferation. As a key protumor chemokine, CCL2 can accelerate tumor progression by recruiting tumor-associated macrophages, promoting angiogenesis, and inducing epithelial-mesenchymal transition.^50^,^51^ The marked decrease in CCL2 levels in the study group after treatment suggests that this combination regimen can further increase the efficacy of conversion therapy by regulating the tumor microenvironment and inhibiting inflammatory chemotaxis, a core advantage over TACE.

In addition, quantitative analysis of tumor morphology and blood supply provides intuitive imaging evidence of conversion efficacy. After treatment, the reduction rate of maximum tumor diameter and tumor necrosis rate were significantly greater in the study group than in the control group, and the decrease in the blood flow signal intensity of tumor-feeding arteries was more pronounced (P<0.05). These findings indicate that the combined regimen can not only achieve significant tumor volume regression but also more thoroughly inhibit tumor blood supply. The mechanism underlying these results may be directly related to the antiangiogenic effect of lenvatinib: its specific inhibition of tumor vascular endothelial cells can reduce blood perfusion in tumor-feeding arteries and, combined with the sustained killing of tumor cells by immune cells, ultimately leads to deep lesion necrosis.^52^ This finding is consistent with the results reported by Wu FD et al.^53^, who reported that the tumor necrosis rate after targeted therapy combined with immunotherapy was significantly greater than that after TACE. This effect may have occurred because although TACE can embolize the main blood-supplying arteries in the short term, some tumors can maintain their blood supply through intrahepatic collateral circulation or neovascularization, resulting in incomplete lesion necrosis.^54^,^55^

There was no significant difference in the overall incidence of adverse reactions between the two groups (P>0.05), confirming that the safety profile of lenvatinib combined with sintilimab is comparable to that of TACE and is well tolerated by patients. All adverse reactions were effectively relieved after symptomatic treatment, and no treatment-related deaths or serious adverse events that significantly affected surgery occurred, further supporting the clinical feasibility of this combination regimen.

### Strengths of the study:

Overall, this retrospective analysis provides more evidence of the efficacy of combining lenvatinib and sintilimab in patients with unresectable HCC. Compared with TACE, this combination strategy resulted in better improvements in the ORR, DCR, and conversion resection rate. Our further analysis revealed that the effects of the combination of lenvatinib and sintilimab were associated with decreased AFP, PIVKA-II, and CCL2 levels. Therefore, we believe that assessing these markers may provide clinical benefits when the most appropriate treatment is selected. However, further research is essential to refine patient stratification and explore biomarkers for predicting treatment response.

### Limitations:

First, as a single-center retrospective study, there may be selection bias in sample enrollment, and the sample size is relatively small; thus, the results should be extrapolated with caution. Second, the follow-up period was relatively short, and key prognostic indicators, such as the postoperative recurrence rate and long-term survival rate, were not analyzed. Finally, the impact of baseline patient characteristics (e.g., tumor differentiation and PD-L1 expression) on therapeutic efficacy was not explored, and no evidence was obtained to inform individualized treatment plans.

## CONCLUSION

Compared with TACE, lenvatinib combined with sintilimab as a conversion therapy for unresectable HCC more effectively improves the objective response rate and conversion-to-resection rate, significantly reduces serum tumor marker levels, and enhances tumor regression and inhibition of tumor blood supply, while demonstrating a safety profile comparable to that of conventional TACE.

### Recommendations:

Future large-sample, multicenter prospective studies with extended follow-up periods are needed to evaluate long-term efficacy. Additionally, studies combining technologies such as genetic testing and radiomics with predictive biomarkers of efficacy may identify a patient population that would benefit most from the combined regimen, enabling precise conversion therapy.

### Author Contributions:

**JL and JZ:** Literature search, study design and manuscript writing. **SC, PO and XL:** Data collection, data analysis and interpretation. Critical Review. **JL and JZ:** Manuscript revision and validation and are responsible for the integrity of the study. All the authors have read and approved the final manuscript.

## References

[1] Chen D, Yang F, Wang XJ, Zhao J (2023). Clinical efficacy of comprehensive rehabilitation intervention and its effect on Quality of Life in patients with Advanced Liver Cancer after Ultrasound-guided Microwave Ablation. Pak J Med Sci.

[2] Villanueva A (2019). Hepatocellular Carcinoma. N Engl J Med.

[3] Moris D, Martinino A, Schiltz S, Allen PJ, Barbas A, Sudan D (2025). Advances in the treatment of hepatocellular carcinoma: An overview of the current and evolving therapeutic landscape for clinicians. CA Cancer J Clin.

[4] Haber PK, Puigvehí M, Castet F, Lourdusamy V, Montal R, Tabrizian P (2021). Evidence-Based Management of Hepatocellular Carcinoma: Systematic Review and Meta-analysis of Randomized Controlled Trials (2002-2020). Gastroenterology.

[5] Takayasu K (2013). Transcatheter arterial chemoembolization for unresectable hepatocellular carcinoma: recent progression and perspective. Oncology.

[6] Xu X, Zhang X, Zhao Y, Zhang X, Tuo H (2025). Comprehensive treatment of intermediate and advanced primary hepatocellular carcinoma based on transcatheter arterial chemoembolization (Review). Oncol Lett.

[7] Yuan Y, He W, Yang Z, Qiu J, Huang Z, Shi Y (2023). TACE-HAIC combined with targeted therapy and immunotherapy versus TACE alone for hepatocellular carcinoma with portal vein tumour thrombus: a propensity score matching study. Int J Surg.

[8] Sangro B, Kudo M, Erinjeri JP, Qin S, Ren Z, Chan SL (2025). Durvalumab with or without bevacizumab with transarterial chemoembolisation in hepatocellular carcinoma (EMERALD-1): a multiregional, randomised, double-blind, placebo-controlled, phase 3 study. Lancet.

[9] Liu LD, Dong SM, Li YY, Nan YM (2023). A clinical study of targeted immunotherapy combined with hepatic arterial chemoembolization in the treatment of liver injury associated with primary liver cancer. Zhonghua Gan Zang Bing Za Zhi Zhonghua Ganzangbing Zazhi Chin J Hepatol.

[10] Wang MD, Xu XJ, Wang KC, Diao YK, Xu JH, Gu LH (2024). Conversion therapy for advanced hepatocellular carcinoma in the era of precision medicine: Current status, challenges and opportunities. Cancer Sci.

[11] Matsuki M, Hoshi T, Yamamoto Y, Ikemori-Kawada M, Minoshima Y, Funahashi Y (2018). Lenvatinib inhibits angiogenesis and tumor fibroblast growth factor signaling pathways in human hepatocellular carcinoma models. Cancer Med.

[12] Capozzi M, Divitiis CD, Ottaiano A, Arx Von C, Scala S, Tatangelo F (2019). Lenvatinib, a molecule with versatile application: from preclinical evidence to future development in anti-cancer treatment. Cancer Manag Res.

[13] Adachi Y, Kamiyama H, Ichikawa K, Fukushima S, Ozawa Y, Yamaguchi S (2022). Inhibition of FGFR Reactivates IFNγ Signaling in Tumor Cells to Enhance the Combined Antitumor Activity of Lenvatinib with Anti-PD-1 Antibodies. Cancer Res.

[14] Wang S, Wang Y, Yu J, Wu H, Zhou Y (2022). Lenvatinib as First-Line Treatment for Unresectable Hepatocellular Carcinoma: A Systematic Review and Meta-Analysis. Cancers.

[15] Hu B, Zou T, Qin W, Shen X, Su Y, Li J (2022). Inhibition of EGFR Overcomes Acquired Lenvatinib Resistance Driven by STAT3-ABCB1 Signaling in Hepatocellular Carcinoma. Cancer Res.

[16] Wysocki PT, Chmiel P, Jassem J, Kraj L (2026). Advancing Systemic Treatment beyond First Line for Advanced Hepatocellular Carcinoma. Curr Oncol Rep.

[17] Fujimoto Y, Namisaki T, Takeda S, Murata K, Enomoto M, Takaya H (2022). Leaky Gut and Severe Adverse Events in Advanced Hepatocellular Carcinoma Treated With Lenvatinib. Anticancer Res.

[18] Ni J, Sun H, Guo G, Zhou X, Zhou Z, Fan R (2026). Combination of programmed death protein-1 inhibitor improves the efficacy of transarterial chemoembolization plus lenvatinib for unresectable intermediate-stage hepatocellular carcinoma: a real-world multi-center retrospective study. Eur J Radiol.

[19] Liu X, Yi Y (2020). Recent updates on Sintilimab in solid tumor immunotherapy. Biomark Res.

[20] Li L, Xu X, Wang W, Huang P, Yu L, Ren Z (2025). Safety and efficacy of PD-1 inhibitor (sintilimab) combined with transarterial chemoembolization as the initial treatment in patients with intermediate-stage hepatocellular carcinoma beyond up-to-seven criteria. J Immunother Cancer.

[21] Cappuyns S, Corbett V, Yarchoan M, Finn RS, Llovet JM (2024). Critical Appraisal of Guideline Recommendations on Systemic Therapies for Advanced Hepatocellular Carcinoma: A Review. JAMA Oncol.

[22] Fulgenzi CAM, D'Alessio A, Airoldi C, Scotti L, Demirtas CO, Gennari A (2022). Comparative efficacy of novel combination strategies for unresectable hepatocellular carcinoma: A network metanalysis of phase III trials. Eur J Cancer.

[23] Cong WM, Bu H, Chen J, Dong H, Zhu YY, Feng LH (2016). Practice guidelines for the pathological diagnosis of primary liver cancer: 2015 update. World J Gastroenterol.

[24] Reig M, Forner A, Rimola J, Ferrer-Fàbrega J, Burrel M, Garcia-Criado Á (2022). BCLC strategy for prognosis prediction and treatment recommendation: The 2022 update. J Hepatol.

[25] Lencioni R, Llovet JM (2010). Modified RECIST (mRECIST) assessment for hepatocellular carcinoma. Semin Liver Dis.

[26] Chang Y, Jeong SW, Young Jang J, Jae Kim Y (2020). Recent Updates of Transarterial Chemoembolilzation in Hepatocellular Carcinoma. Int J Mol Sci.

[27] Jin J, Zhou T, Lou J, Yan S, Wu Y, Xie M (2020). Efficacy of licartin combined with transcatheter hepatic arterial chemoembolization in the treatment of middle-advanced primary liver cancer. J BUON Off J Balk Union Oncol.

[28] Dhondt E, Lambert B, Hermie L, Huyck L, Vanlangenhove P, Geerts A (2022). ^90^Y Radioembolization versus Drug-eluting Bead Chemoembolization for Unresectable Hepatocellular Carcinoma: Results from the TRACE Phase II Randomized Controlled Trial. Radiology.

[29] Lucatelli P, De Rubeis G, Rocco B, Basilico F, Cannavale A, Abbatecola A (2021). Balloon occluded TACE (B-TACE) vs DEM-TACE for HCC: a single center retrospective case control study. BMC Gastroenterol.

[30] Pung L, Ahmad M, Mueller K, Rosenberg J, Stave C, Hwang GL (2017). The Role of Cone-Beam CT in Transcatheter Arterial Chemoembolization for Hepatocellular Carcinoma: A Systematic Review and Meta-analysis. J Vasc Interv Radiol.

[31] Ahmed RM, Ali WA, AbdelHakam AM, Ahmed SH (2024). Detection of hepatocellular carcinoma feeding vessels: MDCT angiography with 3D reconstruction versus digital subtraction angiography. BMC Med Imaging.

[32] Sun L, Xu X, Meng F, Liu Q, Wang H, Li X (2022). Lenvatinib plus transarterial chemoembolization with or without immune checkpoint inhibitors for unresectable hepatocellular carcinoma: A review. Front Oncol.

[33] Li J, Yang B, Teng Z, Liu Y, Li D, Qu X (2024). Efficacy and safety of first-line treatments for advanced hepatocellular carcinoma patients: a systematic review and network meta-analysis. Front Immunol.

[34] Wang D, Zhang L, Yang WH, Zhang LZ, Yu C, Qin J (2025). Arginine-Loaded Nano-Calcium-Phosphate-Stabilized Lipiodol Pickering Emulsions Potentiates Transarterial Embolization-Immunotherapy. Adv Sci (Weinh).

[35] Liu L, Zhuang J, Tan J, Liu T, Fan W, Zhang Y (2022). Doxorubicin-Loaded UiO-66/Bi_2_S_3_ Nanocomposite-Enhanced Synergistic Transarterial Chemoembolization and Photothermal Therapy against Hepatocellular Carcinoma. ACS Appl Mater Interfaces.

[36] Li QM, Sun QC, Jian Y, He JZ, Zhu HB, Hong C (2023). Efficacy and safety of different PD-1 inhibitors in combination with lenvatinib in the treatment of unresectable primary liver cancer: a multicentre retrospective study. Discov Oncol.

[37] Han RY, Gan LJ, Lang MR, Ren SH, Liu DM, Li GT (2024). Lenvatinib, sintilimab combined interventional treatment vs bevacizumab, sintilimab combined interventional treatment for intermediate-advanced unresectable hepatocellular carcinoma. World J Gastroenterol.

[38] Zhao L, Chang N, Shi L, Li F, Meng F, Xie X (2022). Lenvatinib plus sintilimab versus lenvatinib monotherapy as first-line treatment for advanced HBV-related hepatocellular carcinoma: A retrospective, real-world study. Heliyon.

[39] Sun SS, Guo XD, Li WD, Chen JL (2024). Lenvatinib combined with sintilimab plus transarterial chemoembolization as first-line treatment for advanced hepatocellular carcinoma. World J Clin Cases.

[40] Cai M, Huang W, Liang W, Guo Y, Liang L, Lin L (2024). Lenvatinib, sintilimab plus transarterial chemoembolization for advanced stage hepatocellular carcinoma: A phase II study. Liver Int Off J Int Assoc Study Liver.

[41] Lanza C, Ascenti V, Amato GV, Pellegrino G, Triggiani S, Tintori J (2025). All You Need to Know About TACE: A Comprehensive Review of Indications, Techniques, Efficacy, Limits, and Technical Advancement. J Clin Med.

[42] Li W, Lu M, Yuan G, Shi F, Zang M, Hu X (2025). Comparison of the efficacy and safety of TACE-HAIC-MTTs-ICIs and TACE-MTTs-ICIs in the hepatocellular carcinoma: a prognostic analysis based on the dynamic changes of serum AFP. Int J Surg.

[43] Pan Z, Liu D, Cao J, Fu L, Zhang X, Zhu X (2025). China Liver Cancer Study Group Young Investigators (CLEAP). Comparison of Efficacy between Lenvatinib and Bevacizumab in Combination of Immune Checkpoint Inhibitor and Interventional Triple Therapy in Chinese Advanced Hepatocellular Carcinoma: The CLEAP 2302 Study. Liver Cancer.

[44] Liu CF, Zhao XH, Zhu SB, Yu HP, Xing WG, Li HK (2025). Predictors of recurrence after conversion therapy in unresectable hepatocellular carcinoma treated with HAIC, bevacizumab, and sintilimab. Front Immunol.

[45] Wang SY, Su TH, Chen BB, Liu CJ, Liu CH, Yang HC (2022). Prothrombin induced by vitamin K absence or antagonist-II (PIVKA-II) predicts complete responses of transarterial chemoembolization for hepatocellular carcinoma. J Formos Med Assoc.

[46] Do HTT, Lee CH, Cho J (2020). Chemokines and their receptors: multifaceted roles in cancer progression and potential value as cancer prognostic markers. Cancers.

[47] Tian Z, Hou X, Liu W, Shao C, Gao L, Jiang J (2022). Targeted blocking of CCR2 and CXCR2 improves the efficacy of transarterial chemoembolization of hepatocarcinoma. Cancer Cell Int.

[48] Zhao FY, Zhang XM, Qian NS (2025). Two treatment methods for hepatocellular carcinoma: Lenvatinib or bevacizumab combined with sintilimab and interventional therapy. World J Gastroenterol.

[49] Jiang S, Wang Y, Fu X, Lou J, Gong B, Liu W (2025). Clinical significance of AFP and PIVKA-II for predicting prognosis in hepatocellular carcinoma patients treated with immune checkpoint inhibitors. Ther Adv Med Oncol.

[50] Zhang Y, Ding X, Zhang X, Li Y, Xu R, Li HJ (2024). Unveiling the contribution of tumor-associated macrophages in driving epithelial-mesenchymal transition: a review of mechanisms and therapeutic Strategies. Front Pharmacol.

[51] Hajibabaie F, Abedpoor N, Maleki B, Boshtam M, Shenavar N, Zarrabi A (2026). Targeting the CCL2/CCR2 axis in colorectal cancer: Immunopharmacological perspectives and therapeutic strategies. Crit Rev Oncol Hematol.

[52] Une N, Takano-Kasuya M, Kitamura N, Ohta M, Inose T, Kato C (2021). The anti-angiogenic agent lenvatinib induces tumor vessel normalization and enhances radiosensitivity in hepatocellular tumors. Med Oncol.

[53] Wu FD, Zhou HF, Yang W, Zhu D, Wu BF, Shi HB (2025). Transarterial chemoembolization combined with lenvatinib and sintilimab vs lenvatinib alone in intermediate-advanced hepatocellular carcinoma. World J Gastrointest Oncol.

[54] Su J, Kapoor S, Brown DB (2018). Intra- and Extrahepatic Collaterals: Pitfalls and Pathways for Locoregional Therapy. Semin Interv Radiol.

[55] Zhang TQ, Huang ZM, Shen JX, Chen GQ, Shen LJ, Ai F (2019). Safety and effectiveness of multi-antenna microwave ablation-oriented combined therapy for large hepatocellular carcinoma. Ther Adv Gastroenterol.

